# Combined Effects of Vitamin D Status, Renal Function and Age on Serum Parathyroid Hormone Levels

**DOI:** 10.3389/fendo.2021.657991

**Published:** 2021-04-30

**Authors:** Dan Alexandru Niculescu, Laura Georgiana Deacu, Andra Caragheorgheopol, Nicoleta Popescu, Adina Ghemigian, Camelia Procopiuc, Roxana Rosca, Catalina Poiana

**Affiliations:** ^1^ Department of Endocrinology, Carol Davila University of Medicine and Pharmacy, Bucharest, Romania; ^2^ Department of Pituitary and Neuroendocrine Disorders, C. I. Parhon National Institute of Endocrinology, Bucharest, Romania; ^3^ Research Laboratory, C. I. Parhon National Institute of Endocrinology, Bucharest, Romania; ^4^ Biochemistry Department, C. I. Parhon National Institute of Endocrinology, Bucharest, Romania; ^5^ Department of Gonadal Disorders, C. I. Parhon National Institute of Endocrinology, Bucharest, Romania; ^6^ Department of Pediatric Endocrinology, C. I. Parhon National Institute of Endocrinology, Bucharest, Romania; ^7^ Department of Adrenal and Bone Disorders, C. I. Parhon National Institute of Endocrinology, Bucharest, Romania

**Keywords:** parathyroid hormone, 25-hydroxy vitamin D, glomerular filtration rate, chronic kidney disease, age

## Abstract

**Background:**

Vitamin D status and renal function are well-known independent predictors of serum parathyroid hormone (PTH) levels. We aimed to describe the combined effects of 25-hydroxy vitamin D (25(OH)D), glomerular filtration rate (GFR) and age on serum PTH levels across the whole clinical spectrum.

**Methods:**

We retrieved from our endocrinology center database all PTH measurement between 2012 and 2020 for which a simultaneous measurement of serum 25(OH)D, calcium and creatinine was available. Age, sex and diagnosis were available for all subjects. Intact PTH was measured using the same electrochemiluminescence assay.

**Results:**

There were 6,444 adults and 701 children without a diagnosis of hyper- or hypoparathyroidism or abnormal serum calcium levels. In adults with 25(OH)D≥12 ng/mL multiple regression models showed that serum PTH was negatively correlated with both 25(OH)D and GFR. Regression (-0.68 and -1.59 vs. -0.45 and -0.22 respectively), partial correlation (-0.16 and -0.35 vs. -0.12 and -0.10 respectively) and determination coefficients (0.14 vs. 0.031) were higher in CKD than in normal renal function. In subjects with 25(OH)D<12 ng/mL, GFR was the only significant predictor in those with CKD (β-coefficient=-2.5, r=-0.55) and 25(OH)D was the only significant predictor in those with normal renal function (β-coefficient=-2.05, r=-0.11). Increasing age was associated with higher PTH levels only in those with normal renal function and 25(OH)D≥12 ng/mL.

**Conclusions:**

We showed that declining vitamin D and renal function have additive effects on serum PTH in subjects without vitamin D deficiency. In vitamin D deficient subjects this dependency is stronger but is not additive anymore.

## Introduction

Vitamin D is a well-known predictor of serum parathyroid hormone (PTH) levels (1). Numerous studies, including very large samples (2), showed an inverse relation between serum 25-hydroxy vitamin D (25(OH)D) and PTH levels, with a 25(OH)D breakpoint around 10 ng/mL below which the PTH levels rise sharply (3). Partly based on these data, the current guidelines proposed a cut-off value of 10 to 20 ng/mL for vitamin D deficiency (4–6) and 20-30 ng/mL for vitamin D insufficiency (4, 7).

Renal function is another important predictor of serum PTH levels through proximal tubule hydroxylation of 25(OH)D (8) and dependency of 1,25 dihydroxy vitamin D levels on even slight impairment of kidney function (9). Similar to vitamin D studies, various authors found an inverse relation between glomerular filtration rate (GFR) and PTH with a sharp increase below a GFR of 60 mL/min/1.73m^2^ (10, 11). Also, interventional studies showed a significant PTH decrease with vitamin D supplementation in patients with chronic kidney disease (CKD) and vitamin D deficiency or insufficiency (12, 13). Age was also showed to be positively associated with higher PTH levels, independently of vitamin D status (2, 14), but this is probably due to declining renal function with age.

Although some studies that predicted PTH based on 25(OH)D or GFR adjusted for renal function (1) and vitamin D status (10) respectively, the combined effects of both 25(OH)D and GFR across the whole spectrum of possible values are unknown. Our aim was to describe the simultaneous effects of declining renal function and vitamin D and increasing age on serum PTH levels.

## Materials and Methods

### Subjects

We retrospectively retrieved from the electronic database of 3 adult and 1 pediatric endocrinology departments of our institution all PTH measurements between May 20^th^, 2012 and September 30^th^, 2020. For each measurement, we also retrieved the corresponding sex, age, and diagnosis (by the treating physician) and date of measurement. The study was approved by the Institutional Ethic Committee.

There were 17,962 PTH measurements in adult subjects (age>18 years). We excluded 1,550 (8.62%) patients with a diagnosis of primary hyperparathyroidism and 588 (3.27%) patients with a diagnosis of hypoparathyroidism (including pseudohypoparathyroidism) with 15,824 subjects remaining. Of these, 9,831 subjects had a simultaneous measurement of serum 25(OH)D, creatinine and calcium. Of these, 6,931 were made on different subjects and 2,900 were repeated measurements. In those subjects with multiple assessments we used only the first measurement. From the cohort of 6,931 different subjects we excluded 115 (1.65%) with a serum calcium level lower than 8.4 mg/dL and 372 (5.36%) with a serum calcium level over 10.3 mg/dL due to a high probability of an underlying disorder affecting bone and mineral metabolism that was not mentioned in the diagnosis by the treating physician. A total of 6,444 subjects were retained for the analysis, 6,301 not on dialysis and 143 on permanent hemodialysis. Subjects’ characteristics by vitamin D status in the 6,301 sample not on dialysis can be found in **Table 1**.

**Table 1:** Characteristics of adult and pediatric subjects without dialysis by 25(OH)D levels.

	Total	<12 ng/mL	12-20 ng/mL	20-30 ng/mL	>30 ng/mL	p-value
Adults	n=6301	n=977	n=1861	n=2174	n=1289	
Males, n (%)	654 (10.4%)	106 (10.8%)	177 (9.5%)	230 (10.6%)	141 (10.9%)	
Age (years)	59 (49, 66)	59 (49, 67)	58 (49, 66)	58 (49, 65)	60 (50, 66)	0.002
PTH (pg/mL)	44.6 (34.5, 57.0)	52.1 (38.9, 67.7)	45.1 (35.2, 57.5)	43.3 (33.9, 55.0)	41.3 (32.7, 52.3)	<0.001
GFR (mL/min/1.73m^2^)	93.8 (81.8, 102.8)	93.9 (79.8, 103.7)	94.6 (83.1, 103.5)	93.8 (82.6, 102.9)	92.9 (80.3, 101.5)	0.005
GFR category (mL/min/1.73m^2^)						
1 (≥90)	3783 (60.0%)	575 (58.8%)	1147 (61.6%)	1310 (60.2%)	751 (58.3%)	
2 (60-90)	2138 (33.9%)	306 (31.3%)	600 (32.2%)	770 (35.4%)	462 (35.8%)	
3a (45-59)	227 (3.6%)	44 (4.5%)	78 (4.2%)	59 (2.7%)	46 (3.6%)	
3b (30-44)	111 (1.7%)	33 (3.4%)	26 (1.4%)	27 (1.2%)	25 (1.9%)	
4+5 (<30)	42 (0.7%)	19 (1.9%)	10 (0.5%)	8 (0.4%)	5 (0.4%)	
Calcium (mg/dL)	9.5 (9.2, 9.8)	9.4 (9.2, 9.8)	9.5 (9.2, 9.8)	9.5 (9.2, 9.8)	9.5 (9.2, 9.8)	<0.001
Children	n=701	n=64	n=185	n=266	n=186	
Males, n (%)	330 (47.1%)	32 (50.0%)	78 (42.2%)	124 (46.6%)	96 (51.6%)	
Age (years)	12 (8, 14)	13 (12, 15)	13 (10, 15)	12 (8, 14)	7 (3, 12)	<0.001
PTH (pg/mL)	31.1 (23.7, 41.6)	43.2 (32.5, 53.5)	35.6 (28.1, 44.9)	30.6 (23.6, 38.4)	26.4 (20.3, 32.8)	<0.001
Creatinine (mg/dL)	0.5 (0.4, 0.6)	0.6 (0.5, 0.7)	0.6 (0.5, 0.7)	0.5 (0.4, 0.6)	0.5 (0.4, 0.6)	<0.001
Calcium (mg/dL)	9.7 (9.5, 10.0)	9.7 (9.4, 10.0)	9.6 (9.4, 9.9)	9.8 (9.5, 10.0)	9.8 (9.6, 10.0)	<0.001

Data are presented as number (percentage) or as median (25, 75 percentile).

GFR, glomerular filtration rate; PTH, parathyroid hormone.

In the pediatric population there were 1,549 PTH measurements. We excluded 2 (0.12%) patients with a diagnosis of primary hyperparathyroidism and 88 (5.68%) patients with a diagnosis of hypoparathyroidism (including pseudohypoparathyroidism) with 1,459 subjects remaining. Of these, 1,037 subjects had a simultaneous measurement of serum 25(OH)D, creatinine and calcium. Of these, 785 were made on different subjects and 252 were repeated measurements. In those subjects with multiple assessments we used only the first measurement. From the cohort of 785 different subjects we excluded 3 (0.34%) with a serum calcium level lower than 8.4mg/dL and 59 (7.51%) with a serum calcium level over 10.3 mg/dL. GFR could not be calculated in children as height data was not available so we excluded 22 children with serum creatinine ≥0.9 mg/dL. A total of 701 children were retained for the analysis (**Table 1**).

### Biochemistry

Intact PTH was measured by electrochemiluminescence on a Cobas E601 C analyzer (Roche Diagnostics, Indianapolis, IN; with a measuring range 1.2–5000 pg/mL, reference range 15–65 pg/mL, functional sensitivity 6.0 pg/mL, and a variation coefficient of 20%). Serum 25(OH)D was measured by chemiluminescence on a Liaison XL analyzer (DiaSorin, Saluggia, Italy; with a measuring range 4–150 ng/mL, functional sensitivity 4 ng/mL, and variation coefficient of 20%) or by electrochemiluminescence on a Cobas E601 C analyzer (Roche Diagnostics, Indianapolis, IN; with a measuring range 3–70 ng/mL, functional sensitivity 4.01 ng/mL, and variation coefficient of 18.5%).

Calcium was measured by a colorimetric method on a Cobas c 501 analyzer (Roche Diagnostics, Indianapolis, IN; with a measuring range 0.4-20 mg/dL, reference range 8.4-10.3 mg/dL, functional sensitivity 0.4 mg/dL, and a variation coefficient of 1.3%), a Vitros 4600 analyzer (Ortho Clinical Diagnostics, Raritan, NJ; with a measuring range 1-14 mg/dL, reference range 8.4-10.2 mg/dL, functional sensitivity 1 mg/dL, and a variation coefficient of 1.6%) or an Architect C8000 analyzer (Abbott, Abbot Park, IL; with a measuring range of 0.5-24 mg/dL, reference range 8.4-10.2 mg/dL, functional sensitivity 1.0 mg/dL, and a variation coefficient of 1.2%).

Creatinine was measured by a colorimetric method on a Cobas c 501 analyzer (Roche Diagnostics, Indianapolis, IN; with a measuring range 0.17-24.9 mg/dL, functional sensitivity 0.17 mg/dL, and a variation coefficient of 5.0%), a Vitros 4600 analyzer (Ortho Clinical Diagnostics, Raritan, NJ; with a measuring range 0.05-14 mg/dL, functional sensitivity 0.05 mg/dL, and a variation coefficient of 1.8%) or an Architect C8000 analyzer (Abbott, Abbot Park, IL; with a measuring range of 0.05-37 mg/dL, functional sensitivity 0.1 mg/dL, and a variation coefficient of 4.9%).

GFR was calculated using CKD-Epidemiology Collaboration (CKD-EPI) equation (15). Renal function was defined based on GFR according to Kidney Disease Improving Global Outcomes (KDIGO) 2012 Clinical Practice Guideline for the Evaluation and Management of Chronic Kidney Disease (16).

### Statistical Analysis

We tested for normal distribution of age, PTH, GFR and 25(OH)D using D’Agostino-Pearson test. All values were skewed and normal distribution was rejected. All continuous variables are expressed as median (25th, 75th percentiles). Prevalence data are expressed as number (percentage). For comparisons between groups (25(OH)D groups), Kruskal-Wallis test was used.

For univariate and multivariate regression models serum PTH (dependent variable) was log_(10)_-transformed to obtain a normal distribution and the coefficients were then back-transformed. The independent variables in multivariate models were serum 25(OH)D, GFR and sex (0 for females, 1 for males). Age was not introduced in the models because of high correlation (r>0.7) with GFR and multicollinearity. The correlation between 25(OH)D or GFR and PTH is not linear so a breakpoint was used for both 25(OH)D and GFR. Based on ours (14) and others (3) previous work a breakpoint of 12 ng/mL was chosen for 25(OH)D. For GFR a breakpoint of 60 mL/min/1.73m^2^ was chosen (10, 11). A stepwise multiple regression model was fitted for each of the 4 resulting subgroups: (1) 25(OH)D<12 ng/mL and GFR<60 mL/min/1.73m^2^; (2) 25(OH)D<12 ng/mL and GFR≥60 mL/min/1.73m^2^; (3) 25(OH)D≥12 ng/mL and GFR<60 mL/min/1.73m^2^; (4) 25(OH)D≥12 ng/mL and GFR≥60 mL/min/1.73m^2^.

To assess the influence of age on serum PTH ANCOVA was performed within all 4 adult subgroups with log-transformed PTH as the dependent variable, 25(OH)D and GFR as covariates and age group (18-39, 40-59 and >60 years of age respectively) as factor. In children ANCOVA was performed with log-transformed PTH as the dependent variable, 25(OH)D as covariate and age group (0-11 and 12-18 years of age respectively) as factor.

Statistical analysis was carried out using the SigmaPlot 12.5 software (San Jose, CA) and the MedCalc 14.8.1 software (Ostend, Belgium).

## Results

**Figure 1:**
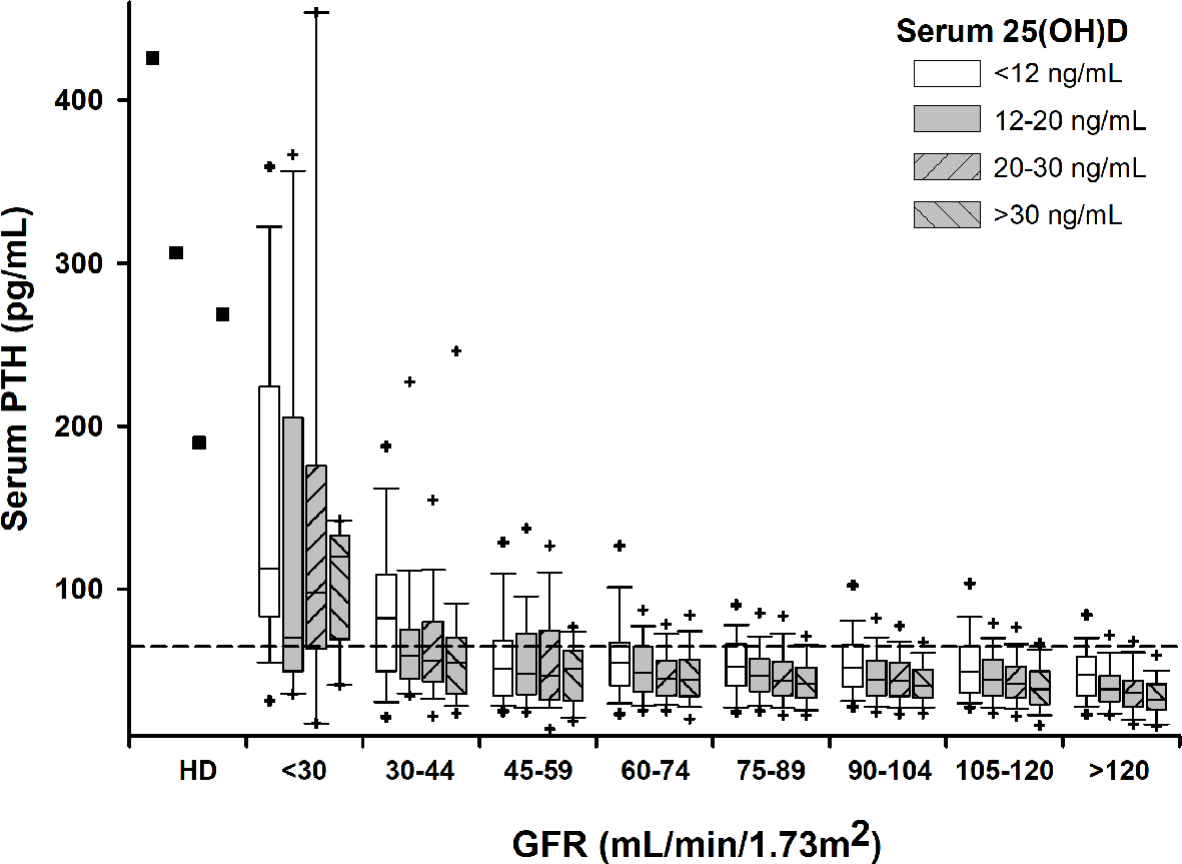
Box-plots for serum PTH by GFR and 25(OH)D intervals. Crosshairs stand for 5^th^/95^th^ percentiles. Squares stand for median values in subjects on permanent hemodialysis (HD). Horizontal dashed line denotes upper normal limit for the PTH assay.

Median PTH rose significantly with decreasing serum 25(OH)D in subjects with normal renal function (GFR≥60 mL/min/1.73m^2^), particularly in vitamin D deficient subjects. The relation between serum PTH and 25(OH)D became less clear in subjects with CKD. Also, PTH rose with decreasing GFR independently of vitamin D status (**Figure 1**). The increase in serum PTH was relatively linear for a GFR over 60 mL/min/1.73m^2^, but the slope became steeper with lower GFR. Interestingly, percentile 95^th^ of serum PTH levels was very close to assay upper limit of normal only for subjects with a GFR over 90 mL/min/1.73m^2^ and a serum 25(OH)D over 30 ng/mL (**Figure 1**). On the other hand, starting with CKD stage 3a, more than 25% of subjects had PTH values above the assay upper limit of normal.

Multiple regression models showed that serum PTH was negatively correlated with both 25(OH)D and GFR in adult subjects without vitamin D deficiency (serum 25(OH)D≥12 ng/mL). Regression, partial correlation and determination coefficients were higher in subjects with CKD, demonstrating a steeper slope and better specification of the model (**Table 2**). In subjects with vitamin D deficiency (25(OH)D<12 ng/mL) GFR was the only significant predictor in those with CKD and 25(OH)D was the only significant predictor in those with normal renal function. Model specification was superior in those with impaired renal function (**Table 2**).

**Table 2:** Regression models predicting serum PTH in adult subjects without hemodialysis.

Serum 25(OH)D (ng/mL)	GFR (mL/min/1.73 m^2^)	Variables in the model	Regression coefficients	r partial	Back-transformed coefficients	R^2^
<12	≥60 (n=881)	constant	1.79		61.65	0.013
		25(OH)D	-0.009	-0.11	-2.05	
	<60 (n=96)	constant	2.31		204.17	0.30
		GFR	-0.011	-0.55	-2.5	
≥12	≥60 (n=5040)	constant	1.79		61.65	0.031
		25(OH)D	-0.002	-0.12	-0.45	
		GFR	-0.001	-0.10	-0.22	
		sex	-0.033	-0.06	-7.31	
	<60 (n=284)	constant	2.19		154.88	0.14
		25(OH)D	-0.003	-0.16	-0.68	
		GFR	-0.007	-0.35	-1.59	

25(OH)D, 25-hydroxy vitamin D; GFR, glomerular filtration rate; PTH, parathyroid hormone; R^2^, coefficient of determination.

The influence of age on serum PTH can be found in **Table 3**. After adjusting for serum 25(OH)D and GFR, age over 40 years was significantly associated with a 5.5 pg/mL increase in serum PTH in adult subjects with normal renal function (GFR≥60 mL/min/1.73m^2^) and no vitamin D deficiency (25(OH)D≥12 ng/mL). In those with vitamin D deficiency and CKD, age was not a significant predictor of PTH anymore. In children without vitamin D deficiency, age over 12 years was associated with a 3 pg/mL significant increase in serum PTH after adjusting for serum 25(OH)D. Children had a significantly lower PTH than adults, irrespective of vitamin D status.

**Table 3:** ANCOVA for the association of serum PTH with age.

Serum 25(OH)D (ng/mL)	GFR (mL/min/1.73 m^2^)	Age group (years)	Mean log-transformed unadjusted PTH	Mean log-transformed adjusted PTH^$^	Back-transformed adjusted mean (95% CI) PTH	p-value
Adults						
<12	≥60 (n=881)	18-39 (n=85)	1.677	1.680	47.9 (43.4-52.9)	NS
		40-59 (n=388)	1.697	1.698	49.9 (47.9-52.1)	NS
		>60 (n=408)	1.723	1.721	52.7 (50.4-55.1)	NS
	<60 (n=96)	18-39 (n=0)	NA	NA	NA	NA
		40-59 (n=20)	1.918	1.855	71.6 (56.1-91.5)	NS
		>60 (n=76)	1.820	1.737	68.7 (60.7-77.8)	NS
≥12	≥60 (n=5040)	18-39 (n=520)	1.566	1.577	37.8 (36.4-39.2)	<0.001*^#^
		40-59 (n =2256)	1.634	1.635	43.2 (42.5-43.9)	<0.001*
		>60 (n=2264)	1.646	1.642	43.9 (43.1-44.6)	<0.001^#^
	<60 (n=284)	18-39 (n=7)	1.663	1.551	35.6 (23.7-53.5)	NS
		40-59 (n=41)	1.780	1.762	57.9 (49.1-68.2)	NS
		>60 (n=236)	1.714	1.721	52.6 (49.1-56.3)	NS
Children						
<12	NA	0-11 (n=29)	1.629	1.629	42.6 (36.9-49.2)	NS
		12-18 (n=35)	1.639	1.639	43.5 (38.2-49.7)	NS
≥12	NA	0-11 (n=396)	1.459	1.466	29.2 (28.1-30.4)	<0.001
		12-18 (n=241)	1.526	1.515	32.7 (31.1-34.4)	<0.001

^$^Adjusted for 25(OH)D and GFR in adults and for 25(OH)D in children.

25(OH)D, 25-hydroxy vitamin D; ANCOVA, analysis of covariance; GFR, glomerular filtration rate; PTH, parathyroid hormone; NA, not available; NS, not significant. *,^#^p < 0.001 between age groups marked with the same symbol.

## Discussion

The effects of vitamin D status, measured by serum 25(OH)D and renal function, measured by GFR, on serum PTH levels have been widely demonstrated (1–3, 10, 11). However, most studies focused only on one of the two predictors either by including subjects with a subset of values (3), by adjusting, as a covariate, for the other variable (10) or by performing univariate modeling (2, 11, 17). As the relation between PTH and both 25(OH)D and GFR is not linear, the results of these studies cannot be extrapolated to the spectrum of all possible values. Our study analyzed the combined effects of vitamin D status and renal function on serum PTH across the whole range of clinically encountered scenarios, from subjects with normal GFR and vitamin D sufficiency to those with severely impaired renal function and vitamin D deficiency.

Our study confirmed the inverse correlation of 25(OH)D and PTH with a steeper increase of PTH at lower serum vitamin D (higher increase in PTH with every unit decrease in 25(OH)D) in patients with normal or mildly decreased renal function (GFR≥60 mL/min/1.73m^2^). The back-transformed correlation coefficients were -0.45 and -2.05 above and below the 12 ng/mL break-point for vitamin D, comparable with the coefficients yielded by studies that included only subjects with normal renal function (3). The same intercept (back-transformed 61.65 pg/mL) for the two models (below and above 12 ng/mL) of subjects without CKD is due to the fact the GFR and sex were kept as independent variables only in one model (no vitamin D deficiency). It is interesting to note that in subjects with normal or mildly decreased renal function about 25% of subjects with vitamin D deficiency (25(OH)D<12 ng/mL) had PTH values above the upper limit of normal while in those with serum 25(OH)D over 30 ng/mL the percent drops to 8%. These figures are equivalent to those found by Valcour (2) and our previous study (14) in subjects under 40 years of age in whom CKD is uncommon.

Interestingly, although median PTH was significantly higher in subjects with vitamin D deficiency compared with subjects with 25(OH)D above 12 ng/mL, the correlation between PTH and 25(OH)D was not significant anymore in subjects with vitamin D deficiency and impaired renal function. This might be due to a lower number of subjects in this category (n = 96) or to the massive effect of decreasing GFR that offsets vitamin D effects.

Our study also demonstrated that GFR is a significant predictor of serum PTH even in subjects with normal or near-normal renal function and no vitamin D deficiency. Although the back-transformed coefficient is small (-0.22, which translates into an increase of serum PTH of 6.6 pg/mL for a 30 ml/min/1.73m^2^ decrease of GFR independently of serum 25(OH)D) our study shows that even a small decrease in renal function, inside the normal range, leads to an increase in serum PTH. The findings of our study are in agreement with those of others (10, 11) who also showed (although only graphical) a minimal effect of GFR on PTH in subjects with normal renal function. Although this relation is clinically non-significant, it raises important questions about the regulation of calcitriol synthesis and action by the kidney (9). Vitamin D deficiency offsets the influence of GFR on serum PTH in subjects with normal or near-normal renal function. In subjects with CKD the influence of GFR on serum PTH is markedly increased (higher beta and correlation coefficients), irrespective of vitamin D status, in agreement with previous studies (10, 11). We also have to note that in subjects with stage 4 CKD less than 25% of subjects have normal PTH levels, even in those with vitamin D sufficiency. Moreover, the variability of PTH serum levels is much higher than in the rest of the population due to some very high values and the strong influence of even subtle changes in GFR.

Although both GFR and 25(OH)D are significant predictors of serum PTH only less than 5% of its variation is explained by these independent predictors in subjects with normal renal function, leaving 95% to random variation or other unexplored factors like calcium intake (18, 19). This is in accordance with some previous studies who found a dependency of PTH on 25(OH)D between 0.044 (17) and 0.066 (1) in subjects with normal renal function. It is important to note that studies reporting very high R^2^ values used mean 25(OH)D for predefined intervals as predictor (2, 3). The predictive value of our models significantly increased in subjects with CKD with up to 30% of PTH variation explained by GFR in those with stage 3a to 4 CKD and vitamin D deficiency. This relatively low dependency of PTH on serum vitamin D and renal function has to be taken into account, particularly in the clinical setting of individual patients, when a metabolic bone disorder is suspected.

Increasing age was frequently associated with higher PTH levels (2, 14, 17, 20). However, it is unclear whether it is an independent effect or it is due to classical confounding effect of lower GFR and 25(OH)D that associates with increasing age (20). Our study showed that age over 40 years is associated with a 5.5 pg/mL increase in serum PTH level independent of GFR and vitamin D compared to 18-39 years of age group in subjects without CKD or vitamin D deficiency. However, this effect is lost in subjects with renal impairment of vitamin D deficiency. Also, in children with normal renal function and no vitamin D deficiency, increasing age was associated with higher serum PTH after adjusting of 25(OH)D levels. These results suggest that, in subjects with normal renal function and no vitamin D deficiency, age is associated with an increase in serum PTH from about 29.2 pg/mL in children under 12 year of age to about 43.5 pg/mL in those over 40 years of age. This increase is independent of serum 25(OH)D and GFR. Whether this increase is physiological or is due to subtle changes in renal function is currently unknown. Moreover, the mechanisms behind it might be different in children and adults.

Taken together, our findings shower that minimum PTH serum levels are found in subject at the high end on normal renal function (GFR over 120 mL/min/1.73m^2^) and vitamin D sufficiency (25(OH)D>30 ng/mL). From this point, PTH increases slowly with decreasing vitamin D and renal function, either alone or combined, until a vitamin D deficiency or CKD stage is reached. Although this small increase is of minimal clinical importance it demonstrates that any subtle change in vitamin D availability or activation leads to compensatory PTH variations. With further deterioration of vitamin D status and renal function PTH rises sharply, frequently to non-physiologic levels. Moreover, these two independent predictors are not additive anymore with GFR being the driving force behind PTH increase.

The main limitation of our study is the possible referral bias as our sample of subjects is hospital-based. However, we tried to minimize this risk by excluding patients with a diagnosis of primary hyperparathyroidism and patients with serum calcium levels below or above the upper limit of normal. Another limitation is the lack of adjustment for active vitamin D treatment (alfacalcidol, calcitriol, vitamin D receptor agonists) as this information was not available. The risk of lower serum PTH values due to active vitamin D treatment is higher in subjects with impaired renal function and might explain the lack of association between 25(OH)D and PTH in subjects with CKD and vitamin D deficiency. However, calcitriol is not available in our country and vitamin D receptor agonists are rarely used in pre-dialysis patients due to reimbursement protocols. Due to lack of height data, GFR could not be calculated in children. We excluded children with serum creatinine over 0.9 mg/dL so we can reasonably presume that the vast majority of children had normal renal function.

The main strength of our study is the large number of subjects, including the pediatric population, that allowed detailed subgroup analysis. There were 977 subjects with vitamin D deficiency (serum 25(OH)D<12 ng/mL) and 380 subjects with CKD. Also, the uniform biochemical assessment (one PTH and only 2 25(OH)D assays) adds to the advantages of our study.

In conclusion, our study provided evidence that vitamin D and renal function have additive effects on serum PTH in those without vitamin D deficiency, even at the higher end of the spectrum. In vitamin D deficient subjects, the dependency of PTH on 25(OH)D and GFR is stronger but is not additive anymore. Age is an independent predictor of PTH only in those without vitamin D deficiency and normal renal function.

## Data Availability Statement

The raw data supporting the conclusions of this article will be made available by the authors, without undue reservation, at written request.

## Ethics Statement

The studies involving human participants were reviewed and approved by Ethics Committee, C. I. Parhon National Institute of Endocrinology, Bucharest, Romania. Written informed consent from the participants’ legal guardian/next of kin was not required to participate in this study in accordance with the national legislation and the institutional requirements.

## Author Contributions

DN collected and analyzed the data, wrote, reviewed and edited the manuscript. LD collected and analyzed the data and reviewed the manuscript. AG performed the PTH and 25(OH)D measurements and reviewed the manuscript. NP performed the calcium and creatinine measurements and reviewed the manuscript. AG, CamP and RR collected the data and reviewed the manuscript. CatP analyzed the data, wrote and reviewed the manuscript. All authors contributed to the article and approved the submitted version.

## Funding

This research received funds for open access publication fees from Carol Davila University of Medicine and Pharmacy.

## Conflict of Interest

The authors declare that the research was conducted in the absence of any commercial or financial relationships that could be construed as a potential conflict of interest.
